# Association between dietary diversity and obesity in the Filipino Women’s Diet and Health Study (FiLWHEL): A cross-sectional study

**DOI:** 10.1371/journal.pone.0206490

**Published:** 2018-11-01

**Authors:** Grace P. Abris, Sherlyn Mae P. Provido, Sangmo Hong, Sung Hoon Yu, Chang Beom Lee, Jung Eun Lee

**Affiliations:** 1 Department of Food and Nutrition, Sookmyung Women’s University, Seoul, Korea; 2 Division of Endocrinology and Metabolism, Department of Internal Medicine, Hallym University Dongtan Sacred Heart Hospital, Gyeonggi, Korea; 3 Division of Endocrinology and Metabolism, Department of Internal Medicine, Hanyang University Guri Hospital, Hanyang University College of Medicine, Gyeonggi, Korea; 4 Department of Food and Nutrition, College of Human Ecology, Research Institute of Human Ecology, Seoul National University, Seoul, Korea; University of Rhode Island, UNITED STATES

## Abstract

**Background:**

Dietary diversity—eating a more varied diet, may be one of the important components of a healthy diet. We aimed to examine whether dietary diversity score was associated with lower prevalence of obesity.

**Methods:**

This is a cross-sectional study of 402 married immigrant participants enrolled in the Filipino Women’s diet and health study (FiLWHEL). Dietary information was obtained using the 24-hour recall method. Anthropometric measurements including height, weight, and waist circumference were directly measured. Dietary diversity score was calculated by summing up the reported number of food groups and additional scores for diversity within food groups were derived. We defined general obesity as body mass index (BMI) of ≥25 kg/m^2^ and abdominal obesity as waist circumference of ≥80 cm. We calculated odds ratios and 95% confidence intervals using the multivariable logistic regression accounting for several potential confounders.

**Results:**

Dietary diversity score was inversely associated with abdominal and general obesity; odds ratios (95% confidence intervals) were 0.49 (0.30–0.82) (p for trend = 0.009) for abdominal obesity and 0.47 (0.28–0.81) (p for trend = 0.008) for general obesity when we compared the third tertile of diversity scores with the first tertile. In the analyses of diversity within food groups, greater diversity in other vegetables was associated with 49% or 50% lower prevalence of abdominal or general obesity, respectively. Furthermore, poultry diversity score was associated with 56% lower prevalence of general obesity.

**Conclusion:**

Our study suggests the evidence that high dietary diversity appears to be related to low prevalence of obesity.

## Introduction

Worldwide obesity is increasing [1] and it has been recognized as a serious public health problem [2] leading to the development of various chronic diseases including type 2 diabetes, cancer, and cardiovascular diseases [3]. Diet contributed to 14.8% of the global burden of diseases and it was estimated that one in every five deaths was due to poor dietary habits [4]. The role of dietary patterns in promoting or preventing obesity and related chronic diseases has drawn greater attention because it reflects the totality of the diet giving a clearer picture of the synergistic effects of the multiple components of the diet [5]. It was suggested that consuming a variety of nutrient-dense food may displace the likelihood of eating energy-dense foods, thus, decreasing body adiposity while individuals enjoy and achieve satiety [6]. Dietary diversity may also be an important component to be considered in terms of chronic disease prevention [7, 8]. Dietary diversity score (DDS) refers to the number of food groups consumed over a given period [9–11]. Studies in Western countries have found that greater dietary diversity, particularly among recommended foods, were favorably associated with biomarkers of health and body weight [7, 12]. Similarly, protective associations have been observed among Iranian populations [13, 14], yet meta-analyses showed high heterogeneity in the association across studies due to differences in methodology, including what is counted toward the DDS, and which populations are studied [15]. Thus, it is difficult to extrapolate the role of DDS in individual populations, and it remains unclear and need to be elucidated.

The meal pattern of Filipinos in the Philippines is usually composed of rice, fish, and meat with small amount of fruits or vegetables [16]. In a study conducted in five developing countries, it was found that Filipino women ranked last in all four food group diversity indicators that had a 15 gram minimum consumption requirement compared to women from Burkina Faso, Mali, Mozambique, and Bangladesh [17]. In our recent study, we have shown that Filipino married immigrants in Korea had lower dietary diversity score and nutrient adequacy compared to Korean women [18]. Also, in South Korea, it was found that Filipino married immigrant women had the highest prevalence of obesity (BMI ≥25 kg/m^2^) [19] compared to their counterpart women from China, Vietnam, or other Asian countries (22%, 16.7%, 7.8%, and 19.2%, respectively) [20]. In like manner, Filipinos in the United States (US) had the highest prevalence of obesity (BMI ≥30 kg/m2) compared to Asian Indian, Vietnamese, and Chinese adults (14%, 6%, 5%, and 4%, respectively) [21, 22].

Given the low dietary diversity and high susceptibility of obesity in the Filipino population, we hypothesized that the lack of dietary diversity could be one of the underlying factors for obesity. To our knowledge, there is no study conducted yet that explores the association between dietary diversity and obesity among Filipino women. Therefore, the current study was designed to examine the association between dietary diversity and general and abdominal obesity.

## Materials and methods

**Fig 1:**
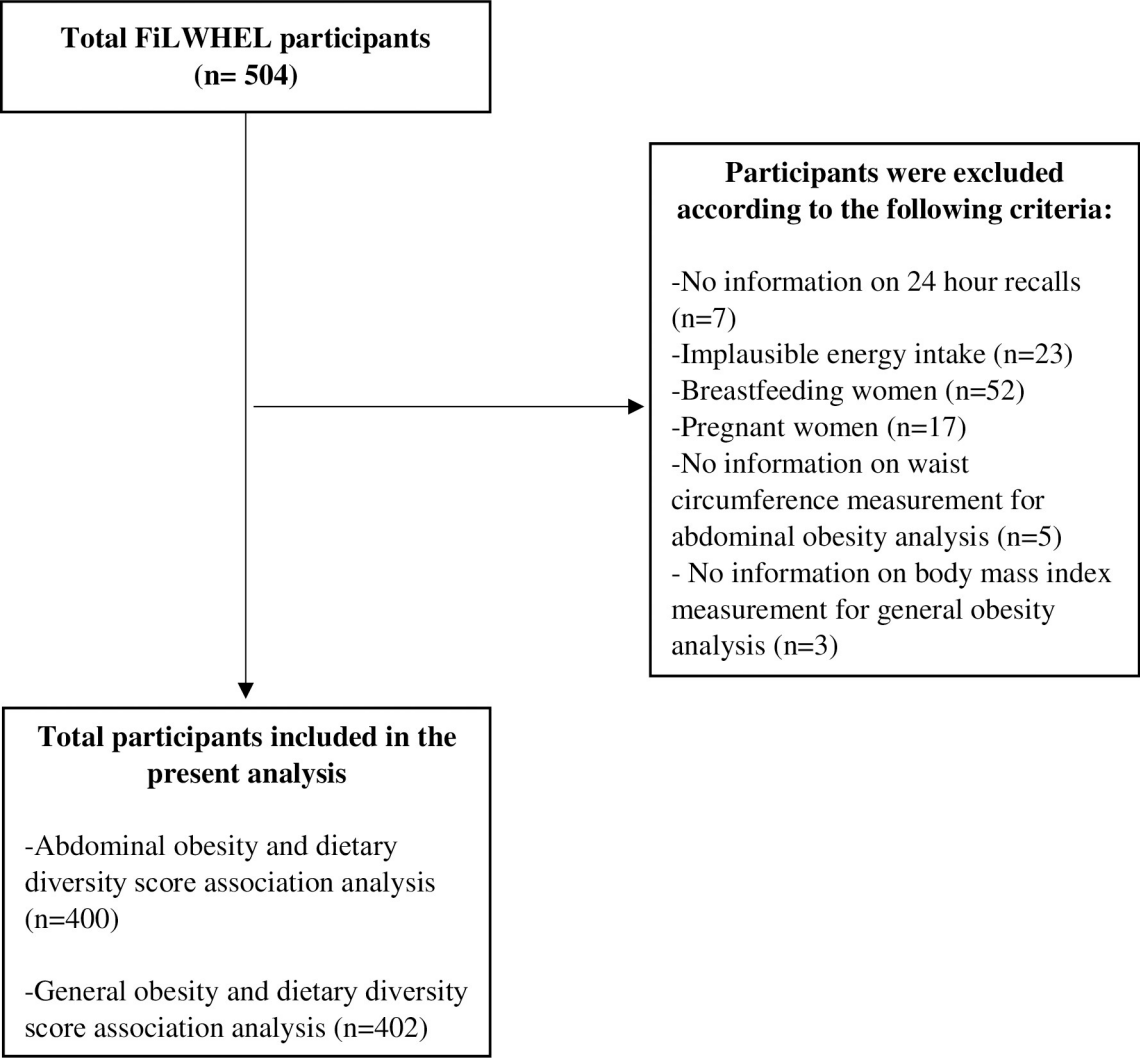
Flow chart of the Filipino women’s diet and health study (FiLWHEL) participants for the dietary diversity score and obesity analysis.

This is a cross-sectional study with data obtained from the Filipino women’s diet and health study (FiLWHEL). FiLWHEL is a cohort of Filipino women aged 19 years or over and are ever married to Korean men in Korea. This cohort has been described elsewhere [23]. FiLWHEL included 504 women who were recruited based on a convenience sampling from different cities in Korea including Seoul, Incheon, and Daejeon and several parts of Gyeonggi and Chungcheong Provinces from March 2014 to April 2016. We disseminated the information through community leaders, personal contacts, and social media in recruiting participants. Among the data collected were information on demographic, socioeconomic, immigration-related questions, diet and other health-related behaviors, and anthropometric measurements including height, weight, and waist circumference. Out of 504 Filipino women enrolled in this study, 497 of them provided dietary information based on a one-day 24-hour recall. The questionnaires were answered through personal interviews for most participants, but in cases where the participants chose to self-administer, assistance was offered whenever needed. The 24-hour recall was conducted solely through face-to-face interview. For the 24-hour recall, we estimated the portion sizes using food miniatures, photographs, household measures, weight/volume, and standard units and portions. The dietary data were entered using the CAN-pro 4.0 (Computer Aided Analysis Program 4.0 for professionals, Korean Society of Nutrition, Seoul, Korea) [24]. When food items were not accessible from the software, we utilized nutrition information from the food composition tables of the Food and Nutrition Research Institute of the Philippines [25] (especially for Filipino food), Korean Rural Development Administration [26], US Department of Agriculture [27], or directly from the manufacturer. For data quality, all interviews throughout the study were conducted by Filipino staff who could speak the Filipino language. All sites followed the same protocol, all study days were supervised with the principal investigators, and all parts of the questionnaire were checked and verified on-site before the participants left. Prior to data coding, questionnaires were checked again and inconsistencies were clarified over the phone and codes were also double-checked. All participants provided written informed consent [23]. This study was approved by the Institutional Review Board of Sookmyung Women's University (reference number SMWU-1311-BR-012). In our present analysis, we excluded participants with implausible energy intake of ≤219.5 kcal or ≥4458.8 kcal per day (> 2 standard deviations above or below the log-transformed mean energy intake), currently pregnant or breastfeeding, missing information on waist circumference or height and weight, providing a sample of 400 or 402 women for the waist circumference or BMI and dietary diversity analysis, respectively (Fig 1).

### Obesity measures

The outcomes were the two types of obesity: abdominal and general obesity. Anthropometric measurements included height, weight, and waist circumference and were directly collected on site in accordance to the standard protocol. General obesity was defined as BMI ≥25 kg/m^2^, calculated from the weight and height, and abdominal obesity was defined as waist circumference ≥80 cm. These cut-offs have been proposed for Asia-Pacific region through the joint effort of the regional office for the Western Pacific of the World Health Organization, the International Association for the Study of Obesity, and the International Obesity task force [19].

### Dietary diversity score

The exposure variable of interest was the dietary diversity score (DDS). DDS was defined as the number of food groups consumed over a 24-hour period [9–11]. The 24-hour recall database of FiLWHEL allowed us to assign ingredients or food items to their appropriate food groups. For example, in the case of mixed dishes, scores were counted toward consumption of more than one food group. In this study, we did not account for serving size but we set the same minimum amount in each food group. Throughout all the analyses, a score of 1 in each food group can be given if a participant at least consumed a minimum of 10 grams [28]. For a few commercial foods, we assigned each food item according to its relevant food group. DDS was based on the 11 food groups: grains and tubers, red meat, poultry, fish, other seafood, legumes/seeds/nuts, egg, dairy, leafy vegetable, other vegetables, and fruits (S1 Table). Other ingredients or food items such as fats and oils, snacks, sweets, other beverages, condiments and seasonings, and other miscellaneous foods were not included in the DDS calculations (S2 Table) [29]. They were excluded for the following reasons: it is not usually feasible to get the right information of oils and fats during recalls, condiments and seasonings may falsely inflate the DDS since they were added in small amounts [29], and besides they were not included in the currently recommended dietary guidelines of Dietary Reference Intakes for Koreans 2015 [30]. A dietary diversity-mortality association study by Kant et al had also similar exclusion criteria of food items [9]. Furthermore, this is also in line with the recommendation set by the FAO and FHI 360 for the minimum dietary diversity for women [29]. The diversity within food groups was examined by counting the number of food items or ingredients in each food group and, for analysis reasons, few food groups were merged.

### Statistical analysis

We divided the DDS groups in tertiles using the RANK procedure in SAS which computes the rank of DDS [31]. Baseline characteristics were compared between tertiles of DDS using means with standard deviations and frequencies. We used analysis of variance (ANOVA) or Kruskal-Wallis test for continuous variables and chi-square test for categorical variables. Multivariable logistic regression analyses were used to estimate ORs and 95% confidence intervals (95% CIs). We examined the change of ORs when deciding which variables to include in the final models. We adjusted for age (continuous, years) and total energy intake (continuous, kcal/d) in model 1. We additionally adjusted for length of stay in Korea (0–4 years, 5–9 years, 10+ years), place of residence (urban, rural), and employment (employed, non-employed) in model 2. Furthermore, we adjusted for parity (no children, one child, two children, three or more children), and history of breastfeeding (continuous, months) in model 3. When we adjusted for alcohol intake (continuous, g/d), education (high school/associate degree or less, college/graduate school), and physical activity (continuous, hours/week), the results did not appreciably change and therefore we did not include these variables. Tests for trends were performed by treating the median value of each DDS tertile as a continuous variable in the model. We also examined the joint association of DDS with obesity prevalence by age (<40 years, ≥40 years), length of residence (<10 years, ≥10 years), alcohol intake (never, ever), smoking (never, ever), vigorous exercise (<1 hour/week, ≥1 hour/week), education (high school/associate degree or less, college/graduate school), yearly income (<10,000,000, ≥10,000,000 won), employment (no, yes), parity (0–1 child, 2 or more children), breastfeeding (never, ever), and change of diet for the past year (no, yes) and performed tests for interactions using the likelihood ratio test. In a sensitivity analysis, we adjusted for energy using the residual method [32] and found that results were similar (data not shown). We also examined the independent relationship of within food group diversity and obesity. All analyses were conducted using SAS 9.4 (SAS Institute Inc., Cary, NC, USA). All statistical tests were two-sided, and P-values <0.05 were considered statistically significant.

## Results

Table 1 shows the baseline characteristics of the study population according to DDS tertiles. Mean age (SD) was similar across tertiles (tertile 1: 35.4 (±8.3); tertile 2: 34.9 (±7.7); and tertile 3: 35.6 (±7.7) (p-value = 0.81)). However, compared with women in the lowest DDS tertile, those who were in the higher tertiles had higher energy intakes (1588.8 kcal/d vs 1762.5 kcal/d and 1949.2 kcal/d, respectively), were less likely to be ever alcohol drinkers (77.4% vs 72.2% and 61.1%, respectively), and had higher proportion of those who had college degree or graduate school (48.0% vs 67.0% and 61.3%, respectively).

**Table 1:** Baseline characteristics according to dietary diversity score in the FiLWHEL (n = 402).

	Dietary Diversity Score			
	Tertile 1	Tertile 2	Tertile 3	*P*-value^1^
Range of DDS	1–5	6	7–10	
Total number of participants	146	92	164	
Age, years	35.4 ± 8.3	34.9 ± 7.7	35.6 ± 7.7	0.81
Energy intake, kcal/d	1588.8 ± 605.6	1762.5 ± 557.8	1949.2 ± 609.9	<0.001
Length of residence, years				0.73
0–4	37 (28.7)	25 (29.8)	35 (21.7)	
5–9	51 (35.4)	29 (34.5)	59 (36.7)	
10+	56 (38.9)	30 (35.7)	67 (41.6)	
Place of residence in Korea, n (%)				0.74
Urban	122 (83.6)	74 (80.4)	138 (84.2)	
Rural	24 (16.4)	18 (19.6)	26 (15.8)	
Alcohol intake, n (%)				0.007
Never	33 (22.6)	25 (27.8)	63 (38.9)	
Ever	113 (77.4)	65 (72.2)	99 (61.1)	
Smoking status, n (%)				0.47
Never	132 (90.4)	80 (88.9)	151 (93.2)	
Ever	14 (9.6)	10 (11.1)	11 (6.8)	
Employment, n (%)				0.12
Non-employed	89 (61.0)	46 (50.6)	82 (50.3)	
Employed	57 (39.0)	45 (49.4)	81 (49.7)	
Education, n (%)				0.007
High school/associate degree or less	76 (52.0)	30 (33.0)	63 (38.7)	
College/graduate school	70 (48.0)	61 (67.0)	100 (61.3)	
Parity, n (%)				0.53
No children	23 (15.8)	21 (23.1)	29 (17.9)	
One child	50 (34.2)	25 (27.5)	62 (38.3)	
Two children	52 (35.6)	33 (36.3)	55 (33.9)	
Three or more children	21 (14.4)	12 (13.2)	16 (9.9)	
History of breastfeeding, months	8.5 ± 12.5	9.8 ± 13.8	9.0 ± 13.1	0.67

Abbreviations: FiLWHEL, Filipino Women’s Diet and Health Study; ANOVA, analysis of variance.

^1^ ANOVA or Kruskal-Wallis test was used for continuous variables and chi-square test was used for categorical variables.

Multivariable-adjusted ORs and 95% CIs for having abdominal and general obesity across DDS tertiles are presented in Table 2. DDS was inversely associated with the odds of being obese and the association remained statistically significant after adjusting for parity and history of breastfeeding for abdominal obesity (OR 0.49; 95% CI 0.30–0.82, p for trend = 0.009) and for general obesity (OR 0.47; 95% CI 0.28–0.81, p for trend = 0.008) when we compared the third tertile of diversity scores with the first tertile.

**Table 2:** Odds ratios (95% confidence interval) of obesity according to dietary diversity score in the FiLWHEL.

	Dietary Diversity Score			
	Tertile 1	Tertile 2	Tertile 3	*P*-trend
Median	4.00	6.00	8.00	
Abdominal Obesity^1^ (WC≥80cm)				
No. of participants	144	92	164	
No. of cases	70	42	58	
Crude Model^2^	1.00	0.89 (0.53–1.50)	0.58 (0.37–0.91)	0.03
Model 1	1.00	0.88 (0.51–1.52)	0.53 (0.32–0.86)	0.02
Model 2	1.00	0.87 (0.51–1.51)	0.52 (0.32–0.86)	0.02
Model 3	1.00	0.85 (0.49–1.47)	0.49 (0.30–0.82)	0.009
General obesity^1^ (BMI≥25kg/m^2^)				
No. of participants	146	92	164	
No. of cases	51	29	40	
Crude Model^2^	1.00	0.86 (0.49–1.50)	0.60 (0.37–0.98)	0.05
Model 1	1.00	0.83 (0.47–1.45)	0.53 (0.32–0.89)	0.02
Model 2	1.00	0.79 (0.45–1.40)	0.51 (0.30–0.86)	0.02
Model 3	1.00	0.75 (0.42–1.35)	0.47 (0.28–0.81)	0.008

Abbreviations: FiLWHEL, Filipino Women’s Diet and Health Study; WC, waist circumference.

^1^ Used the Asia-pacific criteria [19].

^2^ We did not adjust for any variables.

**Model 1:** Adjusted for age (continuous, years) and total energy intake (continuous, kcal/day). **Model 2:** Adjusted for covariates in the Model 1 + years of stay in Korea (0–4 years, 5–9 years, 10+ years), place of residence (urban, rural), and employment (employed, non-employed). **Model 3:** Adjusted for covariates in the Model 2 + parity (no children, one child, two children, three or more children), and history of breastfeeding (continuous, months).

We examined the association between DDS and abdominal and general obesity according to age, length of residence, alcohol intake, smoking status, exercise, education, yearly income, employment, parity, breastfeeding, and change of diet for the past year. We found that these factors did not significantly modify the associations between DDS and abdominal obesity (Table 3), but it was suggestive that the inverse association between abdominal obesity and DDS was limited only to those who have stayed in Korea for ≥10 years (OR 0.32; 95% CI 0.14–0.72, p for heterogeneity = 0.13), never drinkers (OR 0.26; 95% CI 0.10–0.70, p for heterogeneity = 0.13), and to those who did not change their diet for the past year (OR 0.37; 95% CI 0.18–0.75, p for heterogeneity = 0.12) when we compared the third tertile of diversity scores with the first tertile. For general obesity, the inverse association was only limited to women who were never alcohol drinkers (OR 0.19; 95% CI 0.07–0.53, p for heterogeneity = 0.03) (Table 4).

**Table 3:** Odds ratios (95% confidence interval) of abdominal obesity (WC≥80cm)^1^ according to dietary diversity score by other factors in the FiLWHEL (n = 400)^2^.

	DDS tertiles	No. of participants	No. of cases	OR (95 CI)	*P* for hetero-geneity^3^
Age					0.54
<40 years	1	101	41	1.00	
	2	65	24	0.82 (0.43–1.59)	
	3	121	37	0.56 (0.31–1.01)	
≥40 years	1	43	29	1.00	
	2	27	18	1.09 (0.38–3.11)	
	3	43	21	0.47 (0.19–1.18)	
Length of residence					0.13
<10 years	1	87	32	1.00	
	2	54	21	1.09 (0.53–2.23)	
	3	95	29	0.69 (0.36–1.33)	
≥10 years	1	55	38	1.00	
	2	30	16	0.48 (0.18–1.27)	
	3	66	28	0.32 (0.14–0.72)	
Alcohol intake					0.13
Never	1	32	19	1.00	
	2	25	11	0.54 (0.17–1.68)	
	3	63	20	0.26 (0.10–0.70)	
Ever	1	112	51	1.00	
	2	65	30	1.01 (0.53–1.94)	
	3	99	38	0.70 (0.38–1.28)	
Smoking status					0.29
Never	1	130	63	1.00	
	2	80	35	0.84 (0.47–1.50)	
	3	151	52	0.50 (0.30–0.84)	
Ever	1	14	7	1.00	
	2	10	6	1.88 (0.22–16.36)	
	3	11	6	1.37 (0.13–14.12)	
Vigorous exercise					0.56
<1 hour/week	1	122	60	1.00	
	2	75	36	0.98 (0.54–1.80)	
	3	134	48	0.48 (0.27–0.83)	
≥1 hour/week	1	20	9	1.00	
	2	13	4	0.37 (0.07–1.82)	
	3	23	8	0.56 (0.15–2.11)	
Education					0.77
High school/associate degree or less	1	74	34	1.00	
	2	30	12	0.83 (0.32–2.14)	
	3	63	22	0.55 (0.25–1.22)	
College/graduate school	1	70	36	1.00	
	2	61	30	0.95 (0.47–1.95)	
	3	100	36	0.47 (0.24–0.92)	
Household yearly income, Korean won^4^					0.40
<10,000,000	1	50	25	1.00	
	2	26	14	1.31 (0.48–3.59)	
	3	41	20	0.93 (0.38–2.27)	
≥10,000,000	1	65	27	1.00	
	2	40	21	1.60 (0.67–3.83)	
	3	93	32	0.53 (0.25–1.13)	
Employment					0.90
No	1	87	41	1.00	
	2	47	19	0.76 (0.36–1.62)	
	3	83	28	0.52 (0.26–1.01)	
Yes	1	57	29	1.00	
	2	44	23	1.08 (0.47–2.48)	
	3	80	30	0.48 (0.22–1.02)	
Parity					0.24
0–1 child	1	73	32	1.00	
	2	46	22	1.21 (0.56–2.62)	
	3	91	35	0.68 (0.35–1.34)	
2 or more children	1	71	38	1.00	
	2	45	20	0.71 (0.32–1.59)	
	3	71	23	0.40 (0.19–0.85)	
Breastfeeding					0.81
Never	1	47	25	1.00	
	2	30	13	0.84 (0.31–2.31)	
	3	49	21	0.64 (0.27–1.52	
Ever	1	95	44	1.00	
	2	60	29	0.97 (0.49–1.92)	
	3	114	37	0.48 (0.26–0.89)	
Change of diet for the past year					0.12
No	1	78	45	1.00	
	2	50	23	0.61 (0.28–1.30)	
	3	75	29	0.37 (0.18–0.75)	
Yes	1	64	24	1.00	
	2	39	17	1.37 (0.58–3.26)	
	3	86	28	0.80 (0.36–1.78)	

Abbreviations: FiLWHEL, Filipino Women’s Diet and Health Study; WC, waist circumference.

^1^ Used the Asia-pacific criteria [19].

^2^ Adjusted for age (continuous, years), total energy intake (continuous, kcal/day), years of stay in Korea (0–9 years, 10+ years), place of residence (urban, rural), employment (employed, non-employed), parity (0–1 child, 2 or more children), and history of breastfeeding (continuous, months).

^3^ Likelihood ratio test.

^4^ Eighty five participants did not know or had missing information about household yearly income.

**Table 4:** Odds ratios (95% confidence interval) of general obesity (BMI≥25kg/m^2^)^1^ according to dietary diversity score by other factors in the FiLWHEL (n = 402)^2^.

	DDS tertiles	No. of participants	No. of cases	OR (95 CI)	*P* for hetero-geneity^3^
Age					0.61
<40 years	1	101	33	1.00	
	2	65	18	0.73 (0.36–1.48)	
	3	121	26	0.43 (0.22–0.83)	
≥ 40 years	1	45	18	1.00	
	2	27	11	1.07 (0.39–2.96)	
	3	43	14	0.65 (0.26–1.64)	
Length of residence					0.57
<10 years	1	88	25	1.00	
	2	54	14	0.83 (0.38–1.83)	
	3	94	19	0.55 (0.27–1.13)	
≥10 years	1	56	26	1.00	
	2	30	11	0.60 (0.24–1.53)	
	3	67	20	0.41 (0.19–0.92)	
Alcohol intake					0.03
Never	1	33	16	1.00	
	2	25	10	0.63 (0.20–1.95)	
	3	63	12	0.19 (0.07–0.53)	
Ever	1	113	35	1.00	
	2	65	19	0.86 (0.43–1.72)	
	3	99	28	0.72 (0.38–1.37)	
Smoking status					0.21
Never	1	132	45	1.00	
	2	80	24	0.80 (0.43–1.49)	
	3	151	34	0.47 (0.27–0.82)	
Ever	1	14	6	1.00	
	2	10	5	1.67 (0.24–11.70)	
	3	11	6	2.89 (0.35–24.22)	
Vigorous exercise					0.31
<1 hour/week	1	125	44	1.00	
	2	75	25	0.88 (0.47–1.65)	
	3	134	33	0.46 (0.26–0.83)	
≥1 hour/week	1	19	6	1.00	
	2	13	3	0.44 (0.07–2.68)	
	3	23	7	0.72 (0.18–2.98)	
Education					0.53
High school/associate degree or less	1	76	24	1.00	
	2	30	9	0.88 (0.33–2.35)	
	3	63	16	0.66 (0.29–1.48)	
College/graduate school	1	70	27	1.00	
	2	61	20	0.79 (0.37–1.67)	
	3	100	24	0.39 (0.19–0.81)	
Household yearly income, Korean won^4^					0.84
<10,000,000	1	52	19	1.00	
	2	26	8	0.69 (0.23–2.01)	
	3	41	12	0.67 (0.26–1.71)	
≥10,000,000	1	64	20	1.00	
	2	40	17	1.49 (0.62–3.60)	
	3	92	22	0.45 (0.20–1.00)	
Employment					0.87
No	1	89	29	1.00	
	2	46	11	0.61 (0.27–1.42)	
	3	82	19	0.51 (0.25–1.05)	
Yes	1	57	22	1.00	
	2	45	18	1.09 (0.47–2.51)	
	3	81	21	0.47 (0.22–1.04)	
Parity					0.43
0–1 child	1	73	24	1.00	
	2	46	18	1.24 (0.56–2.73)	
	3	91	24	0.60 (0.29–1.22)	
2 or more children	1	73	27	1.00	
	2	45	11	0.55 (0.23–1.32)	
	3	71	16	0.44 (0.20–0.99)	
Breastfeeding					0.40
Never	1	49	20	1.00	
	2	30	10	0.74 (0.27–2.06)	
	3	49	12	0.42 (0.17–1.05)	
Ever	1	95	30	1.00	
	2	60	19	0.94 (0.46–1.94)	
	3	114	28	0.57 (0.30–1.09)	
Change of diet for the past year					0.68
No	1	81	29	1.00	
	2	51	18	0.93 (0.43–2.00)	
	3	75	20	0.52 (0.25–1.09)	
Yes	1	63	22	1.00	
	2	38	10	0.63 (0.25–1.60)	
	3	86	19	0.47 (0.20–1.08)	

Abbreviation: FiLWHEL, Filipino Women’s Diet and Health Study.

^1^ Used the Asia-pacific criteria [19].

^2^ Adjusted for age (continuous, years), total energy intake (continuous, kcal/day), years of stay in Korea (0–9 years, 10+ years), place of residence (urban, rural), employment (employed, non-employed), parity (0–1 child, 2 or more children), and history of breastfeeding (continuous, months).

^3^ Likelihood ratio test.

^4^ Eighty five participants did not know or had missing information about household yearly income.

As shown in tables 5 and 6, the specific food group diversity of other vegetables was inversely associated with the odds of having abdominal and general obesity. Those women who reported the greatest level of diversity in the consumption of other vegetables had a 49% lower odds for abdominal obesity (OR 0.51; 95% CI 0.28–0.91, p for trend = 0.009) (Table 5) and 50% lower odds for general obesity (OR 0.50; 95% CI 0.27–0.94, p for trend = 0.03) compared to women with the least variation of intake. In addition, women who had the higher diversity in the poultry intake had 56% lower odds for general obesity (OR 0.44; 95% CI 0.21–0.90, p for trend = 0.009) compared to those who had zero intake (Table 6). After additionally adjusted for all other specific food group diversity scores, the inverse association of the other vegetables diversity with abdominal obesity and general obesity attenuated but remained statistically significant (p-trend = 0.04) for abdominal obesity and became marginally significant for general obesity (p-trend = 0.08). Furthermore, the inverse association of poultry diversity with general obesity remained statistically significant (p-trend = 0.04) (data not shown).

**Table 5:** Odds ratios (95% confidence interval) of abdominal obesity (WC≥80cm)^1^ according to diversity within food groups in the FiLWHEL (n = 400)^2^.

Food group diversity	n of food groups	Cases/total	OR (95% CI)	*P*-trend
Grain/tuber diversity	1	103/253	1.00	0.23
	2	67/147	1.31 (0.85–2.02)	
Red meat diversity	0	46/102	1.00	0.28
	1	91/206	1.08 (0.65–1.77)	
	2	33/92	0.70 (0.38–1.29)	
Poultry diversity	0	61/122	1.00	0.43
	1	82/216	0.68 (0.42–1.09)	
	2	27/62	0.87 (0.45–1.68)	
Fish and seafood diversity	0	59/144	1.00	0.17
	1	73/149	1.27 (0.78–2.05)	
	2–4	38/107	0.64 (0.37–1.11)	
Legumes/seeds/nuts diversity	0	77/181	1.00	0.72
	1	72/169	0.97 (0.63–1.51)	
	2	21/50	0.87 (0.45–1.71)	
Dairy diversity	0	101/233	1.00	0.69
	1–2	69/167	0.92 (0.60–1.40)	
Leafy vegetables diversity	0	37/84	1.00	
	1	76/171	0.96 (0.55–1.67)	0.17
	2–4	57/145	0.69 (0.39–1.23)	
Other vegetables diversity	0–1	40/84	1.00	0.009
	2	40/85	0.95 (0.51–1.80)	
	3	38/83	0.81 (0.43–1.53)	
	4–10	52/148	0.51 (0.28–0.91)	
Fruits diversity	0	71/161	1.00	0.22
	1	57/133	0.89 (0.55–1.44)	
	2–5	42/106	0.72 (0.42–1.22)	

Abbreviations: FiLWHEL, Filipino Women’s Diet and Health Study; WC, waist circumference.

^1^ Used the Asia-pacific criteria [19].

^2^ Adjusted for age (continuous, years), total energy intake (continuous, kcal/day), years of stay in Korea (0–4 years, 5–9 years, 10+ years), place of residence (urban, rural), employment (employed, non-employed), parity (0–1 child, 2 or more children), and history of breastfeeding (continuous, months).

**Table 6:** Odds ratios (95% confidence interval) of general obesity (BMI≥25kg/m^2^)^1^ according to diversity within food groups in the FiLWHEL (n = 402)^2^.

Food group diversity	n of food groups	Cases/total	OR (95% CI)	*P*-trend
Grain/tuber diversity	1	73/254	1.00	0.63
	2	47/148	1.12 (0.71–1.77)	
Red meat diversity	0	32/105	1.00	>0.99
	1	59/205	0.93 (0.55–1.58)	
	2	29/92	1.01 (0.54–1.89)	
Poultry diversity	0	48/122	1.00	0.009
	1	57/217	0.53 (0.32–0.86)	
	2	15/63	0.44 (0.21–0.90)	
Fish and seafood diversity	0	44/144	1.00	0.19
	1	49/151	0.99 (0.60–1.65)	
	2–4	27/107	0.66 (0.37–1.19)	
Legumes/seeds/nuts diversity	0	55/184	1.00	0.96
	1	49/169	0.93 (0.58–1.49)	
	2	16/49	1.04 (0.52–2.09)	
Dairy diversity	0	70/235	1.00	0.86
	1–2	50/167	0.96 (0.61–1.51)	
Leafy vegetables diversity	0	28/86	1.00	
	1	54/171	0.90 (0.50–1.59)	0.13
	2–4	38/145	0.64 (0.35–1.17)	
Other vegetables diversity	0–1	29/85	1.00	0.03
	2	27/87	0.79 (0.41–1.52)	
	3	28/81	0.89 (0.46–1.73)	
	4–10	36/149	0.50 (0.27–0.94)	
Fruits diversity	0	50/165	1.00	0.64
	1	38/132	0.87 (0.52–1.46)	
	2–5	32/105	0.89 (0.51–1.55)	

Abbreviation: FiLWHEL, Filipino Women’s Diet and Health Study.

^1^ Used the Asia-Pacific Criteria [19].

^2^ Adjusted for age (continuous, years), total energy intake (continuous, kcal/day), years of stay in Korea (0–4 years, 5–9 years, 10+ years), place of residence (urban, rural), employment (employed, non-employed), parity (0–1 child, 2 or more children), and history of breastfeeding (continuous, months).

## Discussion

The present study used a DDS that applies a 10 gram minimum to evaluate the associations between DDS and obesity using BMI and waist circumference. Although energy intake was higher across tertiles of DDS, our study suggested that women who had higher DDS had lower prevalence of abdominal and general obesity compared to those with lower DDS. In particular, we observed an inverse significant association between other vegetables diversity and abdominal and general obesity. Furthermore, poultry diversity was inversely associated with general obesity.

Our results confirm the findings with some of the earlier studies [9, 13]. Using the data from the First National Health and Nutrition Examination Survey (NHANES I) Epidemiologic Follow-up Study (NHEFS) which consisted 10,424 examinees (men: 4,160; women: 6264), Kant et al showed that DDS was inversely associated with BMI among women [9]. In a cross-sectional study among Iranian female youth aged 18–28 years old (n = 289), they found that higher diversity score was associated with lower odds of both general (BMI ≥ 30 kg/m^2^) and abdominal obesity (waist circumference ≥ 88 cm) compared to lower diversity scores [13]. Another cross-sectional study from Iran showed that DDS was inversely associated with metabolic syndrome [33]. However, other studies had also found a positive association between diet diversity and obesity [34, 35]. Results from the subset of Sri Lanka Diabetes Study of 600 adults suggested that DDS was positively associated with obesity using the BMI and waist circumference measures [35]. Another subset from a larger study on nutrition transition and cardiovascular risk which was conducted in Oaxaca, Mexico among 325 Mexican men revealed that DDS increased from underweight to obese groups [34]. Different results could be due to the different methods used in assessing dietary intake and determination of DDS [15]. Variation in the exclusion or inclusion of certain food items may possibly affect the results as what several studies have shown [9, 13, 14, 34, 35]. Also, different population situation could also play a role. In both poor and middle-income countries, DDS could reflect food security [36] or associated also with obesity [35, 37]. Several studies from Western countries found inverse association with several types of cancer [38–43] and type 2 diabetes [8]. In affluent societies, people who have access to healthier foods may tend to have higher dietary variety unlike the challenge of food availability that the developing countries are facing. The reason that we chose this population because Korean diet is varied. The basic meal type of Korean is usually composed of 3 dishes, rice, soup, kimchi and sauce. Though Filipino women are living in Korea it is possible that they still keep their dietary habits from their country of origin, thus higher contrast exists in this group compared to the Korean population. We were able to detect this variation in dietary diversity and found its association with obesity.

In this study, higher diversity in most of the food groups tended to have an inverse association with obesity but in particular, it was observed for the other vegetables and poultry food groups. Similarly, results from the Iranian female youths and from the Tehran Lipid Glucose Study showed that the odds of obesity was significantly lower among those who had the highest vegetable diversity [13, 14]. These inverse associations could be possibly due to the low energy content of vegetables. Our results showed that eating a variety of healthful food is favorably associated with obesity. Furthermore, the inverse relation between DDS and general obesity was stronger among never alcohol drinkers, suggesting a stronger effect among healthier Filipino women. However, we cannot rule out the possibility of false positive chance given the multiple numbers of analyses.

Strengths of our study include the adjustment for potential confounding factors and more specific food groupings. Also, to our knowledge, this is the first study in Korea that examined the association between DDS and obesity among Filipino married immigrant women. However, there are also several limitations that should be considered when examining the results of our study. Firstly, the cross-sectional nature design itself that cannot identify any causal relationships. Secondly, dietary information was based on a single day 24-hour recall that does not reflect usual daily intake. Thirdly, FiLWHEL participants were enrolled based on a convenience sampling from some selected regions in Korea that may limit the generalizability of our findings. Lastly, our findings may still be limited by residual confounding or confounding by unmeasured factors.

## Conclusions

In conclusion, the present findings indicate that higher DDS was associated with lower prevalence of abdominal and general obesity. Our findings support the public health recommendation of eating a more varied healthy diet and also different types of food groups as part of a regular diet for obesity prevention. Given that obesity predisposes various medical condition, further prospective study designs should be conducted to confirm these findings.

## Supporting information

S1 Table Food groups and their subtypes. (DOCX)

S2 Table Food items excluded in the study. (DOCX)
