# Supplementary material for: Association between dietary diversity and obesity in the Filipino Women’s Diet and Health Study (FiLWHEL): A cross-sectional study
Source: PLoS One. 2018 Nov 1;13(11):e0206490. doi: 10.1371/journal.pone.0206490 (PMC6211689; doi:10.1371/journal.pone.0206490)
Supplement: S1 Table — (DOCX) [file pone.0206490.s001.docx]

**S1 Table. Food groups and their subtypes**

| **Food groups** | **Subtypes** |
| --- | --- |
| Grains/tubers | Rice, wheat, barley, corn, millet, sorghum, noodles, bread, potato, sweet potato, and taro |
| Red meat | Beef, pork |
| Poultry | Chicken and duck |
| Egg | Egg |
| Fish | Anchovy, mackerel, and other fish |
| Other seafood | Crustacean shellfish (shrimps, prawns, crabs, lobsters) and molluscan shellfish (abalones, snails, clams, mussels, scallops, oysters, squids, octopus) |
| Legumes/seeds/nuts | Legumes and seeds/nuts |
| Dairy | Cheese, milk, and yogurt |
| Leafy vegetable | Cabbage, other cruciferous, lettuce, spinach, |
|  | and other leafy vegetables |
| Other vegetables | Bean sprouts, peppers and other spices, burdock, carrots, cucumber, laver and other seaweeds, mushroom, pumpkin, radish, tomatoes, and others |
| Fruits | Apple, banana, citrus fruits, grapes, pear, mango, pineapple, strawberry, watermelon, and others |
