# Supplementary material for: Association between dietary diversity and obesity in the Filipino Women’s Diet and Health Study (FiLWHEL): A cross-sectional study
Source: PLoS One. 2018 Nov 1;13(11):e0206490. doi: 10.1371/journal.pone.0206490 (PMC6211689; doi:10.1371/journal.pone.0206490)
Supplement: S2 Table — (DOCX) [file pone.0206490.s002.docx]

**S2 Table. Food items excluded in the study**

| **Food groups** | **Subtypes** |
| --- | --- |
| Fats and oils | Margarine, butter, cream, and different types of oils |
| Snacks | Potato chips, corn chips, biscuits, crackers, and other grain snacks |
| Sweets | Chocolates, candies, cakes, cookies, brownies, pies, wafers, custard, marshmallow, jam, syrups, and jelly |
| Other Beverages | Sodas, chocolate drinks, coffee, tea, energy drinks, and alcohol |
| Condiments and seasonings | Salt, MSG, baking powder, spices, sauces, vinegar, ketchup, broth cubes, and mayonnaise |
| Other miscellaneous foods | Coffee creamer, cream soup powder, coconut milk/powder, and bean paste |
